# Inflammatory Bowel Disease and Colorectal Cancer

**DOI:** 10.3390/cancers16172943

**Published:** 2024-08-23

**Authors:** Jacopo Fanizza, Sarah Bencardino, Mariangela Allocca, Federica Furfaro, Alessandra Zilli, Tommaso Lorenzo Parigi, Gionata Fiorino, Laurent Peyrin-Biroulet, Silvio Danese, Ferdinando D’Amico

**Affiliations:** 1 Department of Gastroenterology and Endoscopy, IRCCS San Raffaele Hospital and Vita-Salute San Raffaele University, 20132 Milan, Italy; fanizza.jacopo@hsr.it (J.F.); bencardino.sarah@hsr.it (S.B.); allocca.mariangela@hsr.it (M.A.); furfaro.federica@hsr.it (F.F.); zilli.alessandra@hsr.it (A.Z.); parigi.tommaso@hsr.it (T.L.P.); sdanese@hotmail.com (S.D.); 2 IBD Unit, Department of Gastroenterology and Digestive Endoscopy, San Camillo-Forlanini Hospital, 00152 Rome, Italy; gionataf@gmail.com; 3 Department of Gastroenterology, Nancy University Hospital, F-54500 Vandœuvre-lès-Nancy, France; peyrinbiroulet@gmail.com; 4 INSERM, NGERE, University of Lorraine, F-54000 Nancy, France; 5 INFINY Institute, Nancy University Hospital, F-54500 Vandœuvre-lès-Nancy, France; 6 FHU-CURE, Nancy University Hospital, F-54500 Vandœuvre-lès-Nancy, France; 7 Groupe Hospitalier Privè Ambroise Parè—Hartmann, Paris IBD Center, 92200 Neuilly sur Seine, France; 8 Division of Gastroenterology and Hepatology, McGill University Health Center, Montreal, QC H4A 3J1, Canada

**Keywords:** inflammatory bowel disease, Crohn’s disease, ulcerative colitis, colorectal cancer, surveillance

## Abstract

**Simple Summary:**

Patients with inflammatory bowel disease (IBD) face an increased risk of colorectal cancer, necessitating a surveillance program to detect early signs of cancer. Evaluating the effectiveness and safety of IBD medications in patients with a history of previous or active cancer, as well as assessing antineoplastic drugs in those with IBD, is crucial. This review outlines known risk factors, surveillance, and appropriate management strategies for patients with IBD and concomitant history of cancer.

**Abstract:**

Patients with inflammatory bowel diseases (IBDs), including both ulcerative colitis (UC) and Crohn’s disease (CD), are at a higher risk of developing colorectal cancer (CRC). However, advancements in endoscopic imaging techniques, integrated surveillance programs, and improved medical therapies have led to a decrease in the incidence of CRC among IBD patients. Currently, the management of patients with IBD who have a history of or ongoing active malignancy is an unmet need. This involves balancing the risk of cancer recurrence/progression with the potential exacerbation of IBD if the medications are discontinued. The objective of this review is to provide an updated summary of the epidemiology, causes, risk factors, and surveillance approaches for CRC in individuals with IBD, and to offer practical guidance on managing IBD patients with history of previous or active cancer.

## 1. Introduction

**Figure 1:**
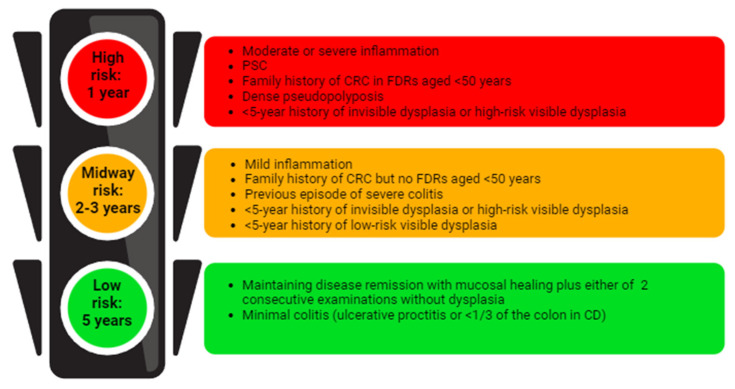
Surveillance strategies recommended for patients with IBD based on risk stratification. CD: Crohn’s disease; CRC: colorectal cancer; FDR: first-degree relative; PSC: primary sclerosing cholangitis.

Inflammatory bowel disease (IBD) refers to chronic, immune-mediated conditions marked by recurring and remitting inflammation of the gastrointestinal tract [1]. Ulcerative colitis (UC) and Crohn’s disease (CD) primarily affect the luminal tract of the gastrointestinal system [2]. However, their exact causes remain unclear and result from a complex interaction of immune dysregulation, microbial imbalance, and environmental factors in individuals with a genetic predisposition [2]. Patients with IBD have a higher risk of developing gastrointestinal cancers, especially colorectal cancer (CRC) [3]. Individuals with long-standing UC and Crohn’s colitis (excluding limited proctitis) face an approximately 2–3-fold increased risk of CRC. This risk varies based on the study, time period, and individual risk factors [2]. Disease duration significantly influences the development of CRC associated with IBD, typically becoming a concern approximately 8 years after the onset of colitis [4]. Although the precise mechanism underlying this association remains unclear, it is plausible that prolonged exposure to repeated cycles of inflammatory insults and epithelial regeneration may contribute to this phenomenon [5]. Other major risk factors include the early onset of IBD in younger individuals, colonic inflammation, the severity of inflammation, backwash ileitis, the presence of strictures, and post-inflammatory polyps [6]. During the natural history of the CRC, it remains uncertain whether the increased risk is due to a higher incidence of dysplastic polyps, which can be challenging to differentiate from benign post-inflammatory polyps during surveillance colonoscopies, or because post-inflammatory polyps are indicative of more severe inflammation [6]. To reduce the risk of CRC, management strategies focus on accurately assessing each patient’s risk and distinguishing between high-risk and low-risk individuals (Figure 1) [2]. Fortunately, there is a trend towards a decline in the rates of CRC over time, likely due to advancements in medical therapies and the implementation of endoscopic screening/surveillance [7].

The aim of this review is to offer an updated summary of IBD-associated colorectal cancer (IBD-CRC), encompassing epidemiology, causes, and risk factors, with a focus on the significance of personalized risk assessment to recommend suitable surveillance protocols. We will also evaluate the efficacy and safety of medical therapies in patients with IBD-CRC and the effect of chemo-radiotherapy on the course of IBD.

## 2. Materials and Methods

We searched PubMed, Embase, and Scopus databases up to 30 June 2024 in order to identify studies regarding CRC and IBD. To achieve this, we employed specific search terms such as “colorectal cancer”, “CRC”, “malignancy”, “neoplasia”, “inflammatory bowel disease”, “IBD”, “Crohn’s disease”, “CD”, “ulcerative colitis”, “UC”, “surveillance”, “screening”, “CRC risk factors”. We limited our search to articles published in English. Three authors (FJ, BS, and DF) reviewed the titles and abstracts to determine eligible studies. The full texts of the selected articles were examined for inclusion, and their reference lists were manually searched to identify any studies that the electronic search might have missed. Abstracts and articles were included based on their relevance.

## 3. Pathogenesis and Epidemiology

IBD patients can develop both sporadic CRC and IBD-CRC [8]. Sporadic CRC typically originates from a dysplastic precursor (usually an adenomatous polyp). It ranks as the third most common cancer in both men and women, with over 1.9 million new cases reported in 2022 [9]. In the development of IBD-CRC, extensive areas of persistently inflamed mucosa are susceptible to neoplastic transformation through a process known as "field cancerization" [2]. The major molecular pathways involved in the development of sporadic CRC, including chromosomal instability, microsatellite instability (MSI), and the CpG island methylator phenotype (CIMP), also play a role in the progression of IBD-CRC [10]. Both types of cancer share common functional driver genes, including APC, P53, MYC, KRAS, PIK3CA, SMAD4, and ARID1A [2]. However, IBD-CRC differs from sporadic CRC in the timing and frequency of genetic alterations. For instance, APC gene mutations and loss are less common and occur later in the dysplasia–carcinoma sequence in IBD-CRC. In contrast, P53 mutations and loss are more frequent and likely happen earlier [3]. Additionally, KRAS and P53 mutations are more prevalent in patients with IBD-CRC than in those without IBD-related dysplasia, making them potential biomarkers for IBD-CRC [3]. 

The global incidence rate of IBD-CRC in CD is estimated to range from 19.5 to 344.9 per 100,000 individuals annually, and from 54.5 to 543.5 per 100,000 individuals annually in patients with UC [11]. Moreover, the incidence varies by geographical location, with higher rates in the US and UK and lower rates in Scandinavian countries [12]. Limited data are available from Asia; however, the prevalence of IBD-CRC is low in this region [13].

For individuals with CD, the risk of IBD-CRC is estimated to be 1.9 times higher [14], whereas for patients with UC, the elevated CRC risk is estimated at a standardized incidence ratio (SIR) of 2.4 [15]. For the entire IBD population, on the other hand, the SIR for IBD-CRC is 1.7 [16,17]. Instead, a recent retrospective study conducted at two tertiary referral centers in Italy reported an annual incidence of CRC among IBD patients of 45 per 100,000 patient-years, and 57 per 100,000 patient-years specifically for UC patients. The SIR for these groups were 1.18 and 1.49, respectively [18]. The risk of IBD-CRC is significantly elevated (by as much as two to three times) in patients with a history of UC and CD of at least 8 years long-standing, referred to as ‘long-standing’ [3]. However, the precise risk can vary depending on different studies and time periods. For example, a meta-analysis from 2001 reported an overall prevalence of CRC in UC patients to be 3.2% [12]. The cumulative risk of CRC was 2% after 10 years, 8% after 20 years, and 18% after 30 years of disease [12]. Conversely, this risk was lower (1%, 3%, and 7% at 10, 20, and 30 years, respectively) in more recent studies [19]. The decrease in CRC risk over time can be credited to improved inflammation management through the use of newer medications and strategies, the introduction of colonoscopic surveillance programs, the optimized visualization of the mucosa using high-definition instruments, the increased adoption of surgical interventions like colectomy and the potential chemopreventive properties of 5-aminosalicylates (5-ASAs) [8,20]. The same reasons, along with other risk factors for CRC (such as obesity, sedentary lifestyle, and high consumption of red and processed meat and fat), may explain the impact of ethnic origin and geographic location on the lower incidence rates observed in some regions [21].

## 4. Risk Factors

To minimize the impact of IBD-CRC, it is crucial to accurately assess each patient’s risk and identify key factors that necessitate frequent monitoring or intensive treatment.

### 4.1. Patient-Related Factors

A family history of CRC is linked to a higher risk of developing IBD-CRC [22]. In fact, patients with first-degree relatives who have CRC face a twofold higher risk of developing CRC compared to those without first-degree CRC relatives [3,23]. Sex differences were also observed, with male patients having a higher risk of CRC than female patients [24], likely due to the protective role of estrogen in CRC development [25]. The most consistently reported risk factors for CRC include primary sclerosing cholangitis (PSC), which is associated with an increased absolute risk of up to 31% [26]. Patients with PSC have a reported fourfold increased risk of developing CRC compared to those without PSC [27]. Finally, being diagnosed with UC at a young age (less than 40 years old) has consistently been linked to a higher risk of developing CRC compared to being diagnosed at an older age [28]. 

### 4.2. Disease-Related Factors

Disease duration is one of the most significant risk factors for IBD-CRC. Although the exact mechanism behind this association is not fully understood, it is possible that the prolonged exposure to repeated cycles of inflammation and epithelial regeneration contributes to the increased risk [5,12]. Extensive disease, typically defined as more than 50% colonic involvement in CD or inflammation extending beyond the splenic flexure in UC at any point during the disease, is linked to a significantly higher risk 2 to 3 times greater of IBD-CRC compared to intermediate-extent CD and left-sided UC [24]. Patients with IBD proctitis have a CRC risk comparable to that of the general population [2]. Histologic inflammation is a more significant risk factor for IBD-CRC than gross endoscopic inflammation. Rutter et al. showed that higher endoscopic and/or histologic inflammation scores were linked to an elevated CRC risk in a multivariate analysis [29]. Other studies [30,31,32] have also confirmed the link between increased severity of endoscopic and histological inflammation and the risk of CRC. It is important to note that an increase in the mean Nancy histologic index was also associated with CRC development [33]. Additionally, patients with IBD who had been previously diagnosed with low-grade dysplasia [5], along with the presence of post-inflammatory polyps or colon stenosis, face a greater risk of CRC compared to those without IBD [24].

## 5. Endoscopic Surveillance

To reduce the risk of CRC, endoscopic surveillance is recommended for patients with UC that extends beyond the rectum and CD with colonic involvement of at least one-third of the colon, beginning 8 years after diagnosis [34]. The intervals for surveillance should be based on previously identified risk factors [34]. The main goal of this surveillance is to detect premalignant lesions that can be removed endoscopically or in early-stage CRC, thereby improving prognosis and treatment outcomes [35]. Figure 1 illustrates the recommended surveillance intervals following the initial colonoscopy, tailored to the individual’s CRC risk.

It is crucial to maintain endoscopic surveillance in patients who have undergone surgical treatment. For instance, those who have had a subtotal colectomy should continue to have their rectum monitored, regardless of whether it is in continuity or diverted [34]. Furthermore, patients who undergo a total proctocolectomy with ileal pouch anastomosis (IPAA) remain at risk for developing CRC due to the retention of a small portion of rectal mucosa at the anastomosis site [34,36]. This risk is a 4.4- and 15.0-fold increase in patients with prior colorectal dysplasia or CRC [37]. There is also a theoretical, albeit extremely rare, risk of developing small bowel cancer within the ileal pouch [34]. Therefore, for patients with high-risk characteristics, such as a history of CRC or dysplasia, PSC, or persistent moderate to severe pouchitis and/or pre-pouch ileitis, annual pouch surveillance is recommended [17,34,38]. 

To enhance the effectiveness of endoscopic surveillance, it is essential to optimize mucosal visualization and improve operator performance [39]. Nonetheless, various international societies offer guidelines on techniques for surveillance colonoscopy [27,38,40,41,42,43,44]. The SCENIC Consensus confirmed that standard definition (SD) white-light endoscopy (WLE) is inferior to high-definition (HD) WLE in detecting dysplasia in patients with IBD [40]. SD endoscopes generate image signals with a resolution ranging from 100,000 to 400,000 pixels, whereas HD endoscopes produce image signals with resolutions of up to one million pixels, equivalent to viewing a surface with 30- to 35-fold magnification [45]. So dye-spray chromoendoscopy (DCE) using methylene blue or indigo carmine or virtual electronic chromoendoscopy (VCE; e.g., iSCAN, narrow band imaging or NBI, blue laser imaging or BLI) with targeted biopsies is suggested in the setting of HD endoscopy [40,46,47] to increase the yield of colonic dysplasia [44]. The use of biopsies during endoscopic surveillance remains a subject of debate. Prior to the adoption of CE, IBD surveillance colonoscopies typically involved taking random biopsies from four quadrants every 10 cm along the colon, in addition to targeted biopsies of any visible lesions [34]. The SCENIC guidelines recommend using either DCE with targeted biopsy sampling or random biopsy sampling with HD-WLE [40]. However, conflicting data have been published regarding the utility and yield of this strategy, primarily due to the wide range of reported dysplasia detected in the random biopsies [48,49]. The subsequent update of the SCENIC guidelines recommends random biopsies only for patients at higher risk, including those with PSC, previous dysplasia, or a scarred and atrophic colon [41]. However, extensive guidance on surveillance colonoscopy techniques is available from various international societies (Table 1). 

Whether conventional colonoscopy is the gold standard for the diagnosis and staging of IBD and for IBD-CRC surveillance, in patients who have had an incomplete or inconclusive colonoscopy or in older, frail or uncooperative patients, computed tomographic colonography (CTC) could be a valid alternative. Systematic reviews indicate that CTC and colonoscopy have comparable sensitivity for detecting CRC and large polyps [50]. However, the sensitivity of CTC is variable and worsens with decreasing polyp size (85%, CI 79–91%, for polyps > 9 mm; for polyps 6–9 mm, 70% sensitivity, CI 55–84%; 48%; 95% CI 25–70%, for polyps < 6 mm) [51].

## 6. IBD Therapy and Cancer

### 6.1. Management of IBD Therapy in Patients with a History of Previous Cancer

In patients with IBD who have a history of cancer, the risk of developing new or recurrent of previous cancer is doubled compared to those without a history of cancer [17]. Due to the relatively small number of IBD patients with a history of cancer, most data on site-specific cancer risks are derived from patients who have undergone organ transplantation or have other immune-mediated inflammatory diseases. Gastrointestinal, bladder, kidney, sarcoma, melanoma, non-melanoma skin cancer, and myeloma are considered high risk for recurrence (>25%) [52,53]. In contrast, lymphoma, testicular cancer, and cervical cancer are associated with a lower risk of recurrence [47,48]. The CESAME study group published the results of a prospective assessment examining the risk of new or recurrent cancer in IBD patients with a history of cancer, whether or not they were receiving immunosuppressants such as thiopurines, methotrexate (MTX), or TNF inhibitors [54]. During follow-up, the incidence of cancer was 21.1 per 1000 patient-years (PY) in patients with a history of cancer, compared to 6.1 per 1000 PY in patients without a history of cancer [54]. Among cancer patients receiving immunosuppressants, 23% exhibited significantly higher rates of new and recurrent neoplastic disease, with incidences of 23.1 per 1000 person-years (PY) and 3.9 per 1000 PY, respectively. This contrasts with the 77% of patients without prior cancer diagnoses, who had lower rates of 13.2 per 1000 PY for new neoplastic disease and 6.0 per 1000 PY for recurrent disease, though the difference was not statistically significant [54]. Comparable findings were reported in a meta-analysis of pooled data on immune-mediated inflammatory diseases (IMIDs) [55] and in a recent multicenter cohort study of consecutive IBD patients with a history of non-digestive neoplasms [56]. Both studies found that using conventional immunosuppressants after an initial tumor does not raise the risk of recurrence or new cancer [57]. The decision to use TNF inhibitors may involve a multidisciplinary approach with oncologists, who will consider the current and recent activity of IBD along with alternative treatment options [17]. A meta-analysis revealed that the use of TNF inhibitors in IMID patients with a history of cancer was not linked to a higher risk of cancer recurrence or the development of new cancers [55]. Comparable results were found in a Spanish registry of IBD patients [58] and in a multicenter study conducted in the United States that examined IBD patients with a history of cancer [59]. In 2020, research was published on the risk of recurrent or new primary cancer among Danish patients with immune-mediated diseases (such as IBD, RA, or psoriasis) who had a history of cancer. This group accounted for 2.8% of CRC cases in these patients [60]. The study found no significant difference in the risk of recurrent or new primary cancer between unexposed patients and those exposed to TNF inhibitor [60]. In addition, there are also long-term data on non-TNF inhibitors. The incidence of cancer among UC patients treated with vedolizumab (VDZ) was less than 1% [61]. The GEMINI long-term safety study similarly found no significant increase in the risk of cancer, suggesting that the gut-selective α4β7 integrin antibody, VDZ, demonstrated a favorable safety profile concerning cancer risk [62]. There are reassuring data also for ustekinumab (UST). In a retrospective study involving IBD patients with a prior history of cancer, UST did not lead to increased rates of new or recurring cancer [63]. Furthermore, safety data on UST at 5 years in CD and 4 years in UC have recently been published [64]. The final cumulative safety data for UST showed that the rates of key safety events, including malignancies, were comparable with placebo [64]. There is a lack of data regarding the risks associated with filgotinib and upadacitinib in patients with a history of cancer. Recently, safety data on long-term treatment with tofacitinib (up to 9.2 years of exposure to the drug) in UC patients did not show an increased risk of cancer [65]. The safety data for IL-23p19 inhibitors primarily come from the use of guselkumab and risankizumab in managing psoriasis. Both risankizumab [66] and guselkumab [67] have shown favorable safety profiles. On the other hand, no long-term safety data are currently available for mirikizumab. In summary, current evidence indicates that advanced therapies do not increase the risk of new or recurrent cancer in IBD patients [44,68].

### 6.2. Management of IBD Therapy in Patients with Current Cancer

Managing IBD patients with CRC or another active cancer is challenging. Similarly, the impact of cancer treatments on the progression of IBD must be considered, as active IBD can complicate treatment choices and influence potential outcomes [69]. In CRC, azathioprine and MTX have been shown to negatively affect both disease-free survival and overall survival [70]. For these reasons, immunosuppressants should be discontinued for IBD patients who are currently diagnosed with or experiencing active cancer until the cancer is controlled [44]. Conversely, there are no restrictions on stopping thiopurine therapy for patients with tumors or pre-neoplastic lesions assessed to have a low risk of recurrence and which have been effectively removed either endoscopically or surgically. However, they should undergo vigilant monitoring for cancer surveillance [44]. Regarding TNF inhibitors, as per the most recent ECCO guidelines, they are deemed suitable for use in patients with IBD who also have concurrent cancer. However, there is a lack of specific data regarding different cancer types and the optimal timing of TNF inhibitors treatment [44]. Similarly, there is a lack of adequate data concerning the safety of VDZ, UST, or JAK inhibitors in patients with active malignancies [44,68]. 

## 7. Management of Chemotherapy, Immunotherapy, and Radiation Therapy in IBD

### 7.1. Chemotherapy

In clinical practice, it is crucial to prioritize cancer treatment in patients with IBD. Conversely, there are limited data available regarding the impact of cancer treatment on the progression of IBD [71,72] and not specific to CRC. In a retrospective study involving 84 patients with IBD who underwent cancer treatment for a solid malignant extraintestinal neoplasm, 66.7% of those with active IBD at the time of their cancer diagnosis achieved remission during their cancer treatment [72]. On the other hand, among IBD patients who were in clinical remission at the time of their cancer diagnosis, 17.4% experienced a flare of IBD following chemotherapy [72]. Cytotoxic chemotherapy may offer potential benefits for IBD patients in inducing or maintaining remission for solid tumors compared to those receiving only hormone therapy or a combination of cytotoxic chemotherapy and adjuvant hormone therapy [72]. In fact, cytotoxic drugs induce cell death or inhibit cell division in rapidly dividing cells, including T lymphocytes and malignant cells, resulting in antitumor and immunosuppressive effects [72]. Similar results were observed in another retrospective study involving 447 patients with IBD and concurrent breast cancer (78%) or prostate cancer (22%) [73]. Quiescent IBD was more likely to relapse in patients who received hormone therapies, either alone (HR 2.00, 95% CI 1.21–3.29) or combined (HR 1.86, 95% CI 1.01–3.43) with cytotoxic therapies [73]. Among the 34 patients who received only cytotoxic chemotherapy, 75% remained in IBD remission for up to 250 months [73]. Although few patients received cytotoxic therapies, there was a trend suggesting that those who underwent cancer treatment monotherapy with cytotoxic agents experienced some protection against IBD relapse [73]. A recent systematic review and meta-analysis, which included 33 studies with a total of 1298 IBD patients undergoing cancer treatment, found that the overall incidence of IBD flares following cancer treatment was 30% [71]. These flares led to the use of systemic steroids in 25% of patients and biological therapies in 10%, while 14% of patients had to discontinue their cancer treatment [71] (Table 2).

### 7.2. Immunotherapy

When administering chemotherapy to IBD patients, it is crucial to exercise caution when interpreting side effects like diarrhea [74]. It is important to determine whether the diarrhea is due to an IBD flare-up [75], a side effect of chemotherapy (such as with 5-fluorouracil and irinotecan) [76], an infectious cause (e.g., clostridium difficile and cytomegalovirus infection), or checkpoint-inhibitor-induced colitis [77]. Immune checkpoint inhibitors (ICIs) target cytotoxic T-lymphocyte-associated protein-4 (CTLA-4) and programmed death-1/ligand (PD-1/PD-L1). ICIs often cause immune-related adverse events (irAEs), with gastrointestinal irAEs being among the most common and typically severe [77]. The risk of gastrointestinal toxicity is 3 times higher in patients with IBD compared to those without IBD [68]. A modified Delphi process was established to reach a consensus on the management of ICI-induced colitis [78]. Oral corticosteroids are considered first-line treatment, with intravenous corticosteroids reserved for cases of severe toxicity [79]. For patients who do not respond to intravenous corticosteroids, advanced therapy could be an off-label option. In fact, in this context, infliximab (IFX) has been shown to be safe for use in patients with an active tumor and coexisting ICI-induced enterocolitis [80]. Some case series have also demonstrated the potential usefulness of VDZ [81] and tofacitinib [82] in this context. In this context, it is important to remember that the occurrence of irAEs is also less frequent after immunotherapy than after chemotherapy [83]. A systematic review and meta-analysis conducted by J. Meserve et al. in 2020 identified 12 studies that examined the impact of immune checkpoint inhibitors on patients with IBD [84]. In total, 40% of patients experienced an IBD relapse with immune checkpoint inhibitor therapy. While data on predictors of IBD relapse were limited, the largest retrospective analysis in the meta-analysis found that CTLA-4 inhibition was associated with an increased risk of gastrointestinal irAEs [84]. Similar results have been observed in another study [85]. However, while blocking the PD-1/PD-L1 pathway has shown significant therapeutic benefits for various cancers, the effectiveness of ICIs in treating CRC is limited, benefiting only a small subset of patients [83]. Currently, pembrolizumab and nivolumab are the only PD-1-blocking antibodies that have shown effectiveness in treating patients with metastatic colorectal cancer (CRC) characterized by mismatch repair deficiency (dMMR) and high microsatellite instability (MSI-H). This has led to their swift approval by the Food and Drug Administration (FDA) [86]. As a result, limited data are available on patients with both IBD and CRC, making it difficult to assess the risk of disease progression after starting therapy with ICIs. 

### 7.3. Radiotherapy

Data on the use of radiotherapy in patients with IBD are limited. Most oncologists avoid pelvic radiotherapy in IBD patients because of historically documented higher risks of toxicities and disease flares [87,88]. In a retrospective study of IBD patients with prostate cancer, 47% received radiotherapy. Among these patients, 10.6% experienced increased IBD flare-ups compared to 5.7% in those who did not receive radiotherapy, both within 6 months and 6–12 months after treatment (4.3% vs. 1.9%) [89]. Additionally, another study found that the 5-year survival rate for rectal cancers in patients with IBD who received pelvic radiation was similar to that of patients without prior IBD, with no increase in gastrointestinal toxicity observed [90]. Additionally, a systematic review showed that radiotherapy is generally safe for IBD patients, with acceptable toxicity profiles and a similar incidence of flares between those treated with radiotherapy and those who underwent surgery [91]. This indicates that radiotherapy is not an absolute contraindication for patients with IBD [88] (Table 2).

**Table 2:** Main results of some studies concerning the pharmacological and non-pharmacological treatment of active cancer in patients with IBD. IBD, inflammatory bowel disease; GI, gastrointestinal; UC, ulcerative colitis; CD, Crohn’s disease; IBD-U, IBD-unclassified; CT, chemotherapy; HR, hazard ratio; XRT, radiotherapy; ADT, androgen deprivation therapy.

Study, Authors, Year of Publication	Patients and IBD Subtype (IBD Remission or Active IBD, if Available)	Type of Cancer (Type of Cancer in IBD Remission, Type of Cancer in Active IBD)	Typer of Cancer Treatment (Type of Treatment in IBD Remission, Type of Treatment in Active IBD)	Main Results		
Effects of Cancer Treatment on IBD Remission and Reactivation, Jordan E. Axelrad et al., 2012 [72]	84 patients - UC 45 (40, 5) - CD 39 (29, 10)	Breast 37 (30, 7) Lung 12 (10, 2) GI 19 (16, 3)	Cytotoxic CT 46 (41, 5) Hormonal 22 (16, 6) Cytotoxic + hormonal 16 (12, 4)	Active IBD group 10 IBD remission: 5 cytotoxic CT 1 hormonal 4 combination therapy	Inactive IBD group 12 IBD flare-ups: 1 cytotoxic CT 6 hormonal 5 combination therapy	
Hormone Therapy for Cancer is a Risk Factor for Relapse of IBD, J. E. Axelrad et al., 2021 [73]	447 patients - UC 238 (214, 24) - CD 197 (175, 22) - IBD-U 12 (11, 1)	Breast 346 (315, 31) Prostate 101 (85, 16)	Cytotoxic CT 34 (34, 0) Hormonal 187 (164, 23) Cytotoxic CT + hormonal 73 (65, 8) Other therapies or unknown 165 (148, 17)	Active IBD group, risk for IBD remission (95% CI) Cytotoxic CT: - Hormonal: HR 1.98 (0.42–9.34) Cytotoxic CT + hormonal: HR 2.09 (0.35–12.5)	Inactive IBD group, risk of flare-up (95% CI) Cytotoxic CT: HR 0.91 (0.34–2.42) Hormonal: HR 2.00 (1.21–3.29) Cytotoxic CT + hormonal: HR 1.86 (1.01–3.43)	
Acute and late toxicity of patients with IBD undergoing irradiation for abdominal and pelvic neoplasm, C. G. Willett et al., 1999 [87]	28 patients - UC 18 - CD 10	CRC 17 Prostate 7 Endometrial 2 Pancreatic 1 Small bowel 1 [no data regarding active or remission IBD]	Radiotherapy techniques Conventional 12 Specialized 16 [no data regarding active or remission IBD]	Frequency of toxicities (conventional, specialized) Total severe toxicity 46% (58%, 38%) - Severe acute toxicity 21% (17%, 25%) - Severe late toxicity 29% (50%, 13%)		
Rates of Adverse IBD-Related Outcomes for Patients with IBD and Concomitant Prostate Cancer Treated With Radiation Therapy, L. A. Feagins et al., 2020 [89]	100 patients - UC 66 - CD 29 - IBD-U 5	Prostate 100	XRT/brachytherapy 47 Nonradiation therapy 53	Rates of IBD flare-up XRT/brachytherapy vs. nonradiation therapy - within 6 months: 10.6%, 5.7% - within 6–12 months: 4.3%, 1.9% - within 12–24 months: 8.5%, 9.4%		
Implications of prostate cancer treatment in men with IBD, P. S. Kirk et al., 2018 [91]	205 patients [no data regarding IBD type]	Prostate 205	Surgery 85 Radiotherapy 56 ADT/observation 64	Rate of IBD flare-up in years following treatment		
				Surgery 13% Radiotherapy 23% *p* = 0.28	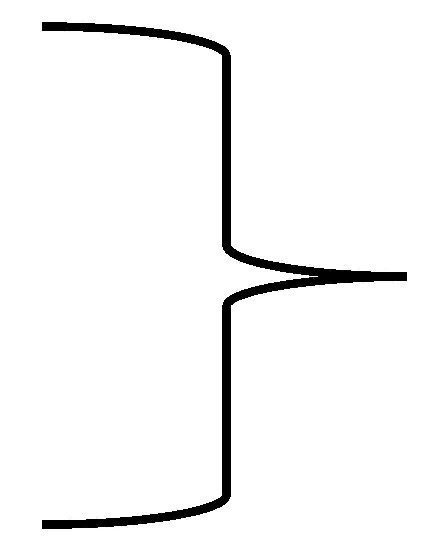	ADT/observation 19%

Table 3 Includes ongoing studies related to some of the topics discussed.

## 8. Discussion

Patients with CD and UC have a higher risk of developing CRC compared to the general population [3]. Despite well-documented risk factors [5], the availability of guidelines for proper screening, surveillance, and management of pre-cancerous lesions [34,38,40], and advancements in endoscope fidelity and imaging techniques, its prognosis remains poorer than that of sporadic CRC [3]. Advancements in disease management, technology, and endoscopic quality have significantly altered our perception of precancerous lesions in IBD, bringing it closer to how we view dysplasia in the non-IBD population [38]. The practice of performing non-targeted biopsies and recommending colectomy for patients with low-grade or invisible dysplasia is increasingly being challenged. Instead, there is a growing preference for approaches that focus on meticulous inspection and targeted sampling of visible and subtle lesions using for example HD-WLE and DCE [38]. While endoscope fidelity and imaging techniques continue to advance, alternatives like the colon capsule are also developing. Capsule imaging is now employed to assess CD, although no publications currently address its use in detecting dysplasia in IBD [92]. Additionally, capsule imaging cannot perform biopsies, and it is well documented that predicting IBD lesions histologically from visual images is suboptimal [34]. Artificial intelligence (AI)-based detection algorithms represent an exciting new frontier with the potential to assist endoscopists in identifying IBD dysplasia. AI capabilities have been tested for detecting colorectal neoplasia, but not specifically in patients with IBD [93]. In the future, AI could become an effective and safe tool for enhancing the quality of examinations in real time (e.g., sampling time, control of blind spots), potentially increasing the adenoma detection rate even in IBD patients [94]. Regarding patients with a previous or recent cancer diagnosis, there are limited data on the efficacy and safety of IBD therapies in patients with CRC. The recommendations in the latest ECCO guidelines for managing patients with IBD who have a history of or active oncological conditions are primarily based on data from other autoimmune diseases or various oncological situations [44,95,96]. For these reasons, the treatment of IBD patients following a cancer diagnosis necessitates a personalized approach. One of the most challenging and increasingly common areas of decision-making involves patients with a recent or active malignancy [44]. Therefore, if there is an active cancer, the primary focus should be on determining the appropriate cancer treatment under the guidance of a multidisciplinary team including an oncologist, gastroenterologist, surgeon, radiologist [69]. Having a multidisciplinary team is crucial for effective therapy management, as it enhances the quality of care, patient satisfaction, and overall outcomes [97]. Additionally, the appropriate therapy must be tailored to the patient’s characteristics, risk related to both IBD (such as disease severity, activity, choice of drug and risk of flare-up) and cancer (including risk of recurrence and time since cancer resolution) [68]. For these patients, it is also crucial to assess whether to start a new therapy or continue an existing IBD treatment in the event of a cancer diagnosis, especially if the IBD is active [44]. In the former scenario, selecting a treatment with the most favorable safety profile, including indirect data from non-IBD populations where relevant, is generally advisable. Regarding existing IBD treatments in the context of active cancer, it is recommended to withhold immunosuppressive therapies, particularly thiopurines [44]. For patients already receiving TNF inhibitors, treatment can be continued if the IBD status risk assessment indicates a high risk of flare [44]. When patients experience flare-ups during active cancer treatment, other causes such as infections or diarrhea resulting from treatment with ICIs should first be ruled out [68]. After differential diagnosis, 5-ASA and corticosteroids are considered safe and can be used as first-line treatments for naive patients with clinically active IBD. If there is no response to corticosteroids or in patients already treated with advanced therapies, VDZ, UST, or selective IL-23 inhibitors may be considered viable alternatives [68]. TNF inhibitors should be considered in patients with severe disease activity [68]. Therefore, prioritizing survival from cancer, it is reasonable to continue cancer therapy with close monitoring for IBD using non-invasive tools such as intestinal ultrasound [98] and fecal calprotectin measurement [99]. 

## 9. Conclusions

Patients with IBD have a higher risk of developing CRC, making it crucial to identify risk factors and high-risk individuals to enhance their prognosis through targeted surveillance programs. The introduction of advanced imaging techniques has significantly enhanced the detection of precancerous lesions. In patients with IBD and concomitant active cancer or history of cancer, it is crucial to balance the benefit–risk ratio of IBD medications before making therapeutic decisions. An individualized approach involving a multidisciplinary team and patient preferences is essential to achieve optimal disease control. Despite significant progress in diagnostics and treatment, several questions remain unresolved. These include the optimal management of pseudopolyps, the effectiveness of random biopsies during HD-WL colonoscopy, the safety of extending surveillance intervals for low-risk populations, and the ideal disease duration before initiating dysplasia surveillance. Additionally, further research is needed to enhance our understanding of the mechanisms driving IBD-CRC and to discover new therapeutic targets and interventions that could reduce carcinogenesis risk, including through clinical trials involving CRC patients.

## Figures and Tables

**Table 1:** Overview of recommendations from international organizations for dysplasia surveillance in patients with IBD. ACG, American College of Gastroenterology; AGA, American Gastroenterological Association; BSG, British Society of Gastroenterology; CE, chromoendoscopy; ECCO, European Crohn’s and Colitis Organization; HD, high definition; SD, standard definition; WLE, white-light endoscopy; NBI, narrow band imaging; PSC, primary sclerosing cholangitis; VCE, virtual chromoendoscopy.

Guideline (Year of Publication)	Type of Endoscopic Surveillance	Random or Targeted Biopsies
SCENIC Consensus (2015) [40]	HD recommended If SD, dye-CE recommended If HD, dye-CE suggested	No consensus
SCENIC commentary (2022) [41]	HD-WLE, dye-CE, or VCE	Random limited to highest-risk groups only (PSC, prior dysplasia, atrophic scarred colon, ongoing active inflammation)
ECCO Guideline (2017) [27]	HD recommended	Random if WL Targeted only if dye-CE
ECCO Guideline (2023) [44]	HD-WLE, dye-CE, or VCE	Targeted biopsies Random in high-risk (PSC or history of dysplasia)
ACG Clinical Guideline (2019) [43]	If SD, dye-CE recommended If HD, dye-CE or VCE recommended	No recommendation
AGA Clinical Practice update (2021) [38]	HD recommended Dye-CE should be considered VCE acceptable alternative if HD	Random if WL only and all patients with high risk (PSC or history of dysplasia) Targeted if dye-CE or VCE
BSG Guideline (2019) [42]	HD recommended If SD, dye-CE recommended If HD, dye-CE suggested NBI not suggested	Targeted recommended

**Table 3:** Some ongoing studies concerning IBD patients and the risk associated with CRC. IBD, inflammatory bowel disease; CRC, colorectal cancer; HD, high definition; WLE, white-light endoscopy; CE, chromoendoscopy; UC, ulcerative colitis; CD, Crohn’s disease; PSC, primary sclerosing cholangitis; IBD-U, inflammatory bowel disease unclassified; LCI, linked color imaging.

Study Title	Aim of the Study	Patients	NCT Number
IBD neoplasia surveillance pilot RCT (IBD Dysplasia)	Random and targeted biopsies vs. targeted biopsies alone for CRC screening in adult persons with colonic IBD	600 with long-standing CD or UC or any disease duration in case of PSC	NCT04067778
Back-to-back endoscopy versus single-pass endoscopy and CE in IBD surveillance (HELIOS)	Back-to-back HD-WLE vs. single-pass HD-WLE vs. CE	563 with long-standing CD or UC or any disease duration in case of PSC	NCT04291976
HD colonoscopy vs. dye-spraying chromo-colonoscopy in screening patients with long-standing IBD	HD colonoscopy vs. dye-spraying chromo-colonoscopy	500 (estimated) with long-standing CD, UC, or IBD-U or any disease duration in case of PSC	NCT04191655
LCI vs. WLE for colorectal dysplasia in UC	LCI vs. conventional colonoscopy using WL for detection of colorectal dysplasia in UC	60 (estimated) with UC	NCT02772406
Dysplasia in inflammatory chronic idiopathic colitis long-standing	Assess the incidence of dysplasia and CRC in patients with chronic idiopathic inflammatory colitis treated with biologics, mesalamine, and immunosuppressive drug combinations	300 (estimated) with long-standing CD or UC	NCT03096717
Testing atorvastatin to lower colon cancer risk in long-standing UC	To determine the effect of atorvastatin treatment on reducing the fraction of colonic epithelial cells expressing mutant p53 protein	70 (estimated) with long-standing UC	NCT04767984

## Data Availability

Data sharing is not applicable to this article as no new data were created or analyzed in this study.
